# A novel mechanoeffector role of fibroblast S100A4 in myofibroblast transdifferentiation and fibrosis

**DOI:** 10.1016/j.jbc.2023.105530

**Published:** 2023-12-10

**Authors:** Brian D. Southern, Haiyan Li, Hongxia Mao, James F. Crish, Lisa M. Grove, Rachel G. Scheraga, Sanaa Mansoor, Amanda Reinhardt, Susamma Abraham, Gauravi Deshpande, Alicia Loui, Andrei I. Ivanov, Steven S. Rosenfeld, Anne R. Bresnick, Mitchell A. Olman

**Affiliations:** 1 Lerner Research Institute Department of Inflammation and Immunity, Cleveland Clinic, Cleveland, Ohio, USA; 2 Respiratory Institute, Cleveland Clinic, Cleveland, Ohio, USA; 3 Lerner Research Institute Imaging Core, Cleveland Clinic, Cleveland, Ohio, USA; 4 University of Pittsburgh, Pittsburgh, Pennsylvania, USA; 5 Division of Hematology/Oncology, Mayo Clinic Jacksonville, Jacksonville, Florida, USA; 6 Department of Biochemistry, Albert Einstein College of Medicine, Bronx, New York, USA

**Keywords:** fibrosis, cytoskeleton, mechanotransduction, myofibroblast differentiation, S100A4

## Abstract

Fibroblast to myofibroblast transdifferentiation mediates numerous fibrotic disorders, such as idiopathic pulmonary fibrosis (IPF). We have previously demonstrated that non-muscle myosin II (NMII) is activated in response to fibrotic lung extracellular matrix, thereby mediating myofibroblast transdifferentiation. NMII-A is known to interact with the calcium-binding protein S100A4, but the mechanism by which S100A4 regulates fibrotic disorders is unclear. In this study, we show that fibroblast S100A4 is a calcium-dependent, mechanoeffector protein that is uniquely sensitive to pathophysiologic-range lung stiffness (8–25 kPa) and thereby mediates myofibroblast transdifferentiation. Re-expression of endogenous fibroblast S100A4 rescues the myofibroblastic phenotype in S100A4 KO fibroblasts. Analysis of NMII-A/actin dynamics reveals that S100A4 mediates the unraveling and redistribution of peripheral actomyosin to a central location, resulting in a contractile myofibroblast. Furthermore, S100A4 loss protects against murine *in vivo* pulmonary fibrosis, and S100A4 expression is dysregulated in IPF. Our data reveal a novel mechanosensor/effector role for endogenous fibroblast S100A4 in inducing cytoskeletal redistribution in fibrotic disorders such as IPF.

Fibrotic disorders account for approximately 45% of deaths in the United States (1). Fibroblast activation and myofibroblast transdifferentiation play critical roles in the pathogenesis of fibroproliferative diseases of the lung, skin, kidney, heart, liver, and vasculature as well as in mediating cancer-stromal cell interactions (2, 3, 4, 5, 6, 7, 8, 9, 10, 11). Our work and that of others have improved our understanding of how environmental cues, such as extracellular matrix (ECM) stiffness, can drive the pro-fibrotic phenotype and create a feed-forward cycle of progressive tissue fibrosis (12, 13, 14, 15). It has been demonstrated using atomic force microscopy that normal lung stiffness ranges from 0.5 to 3 kPa (Young’s Modulus), whereas lung tissue stiffness increases 10-fold (3–25 kPa; Young’s modulus) in murine and human fibrotic lung lesions (16, 17, 18). Current anti-fibrotic therapies focus on known pro-fibrotic soluble mediator signals but fail to halt or reverse established fibrosis (19, 20, 21). It is critical that the dynamic cell-matrix interactions that perpetuate fibrosis are better understood so that novel therapies can be developed to halt the inexorable, fatal progression of fibrosis (22).

Non-muscle myosin II (NMII) is a contractile protein that generates force in non-muscle cells by binding with actin filaments (23). Using a novel assay system that allows analysis of fibroblast behavior in response to actual normal and fibrotic lung tissue, we previously published that lung ECM biophysical cues can drive pro-fibrotic fibroblast phenotypes (17). Specifically, our data indicate that the stiffened ECM of fibrotic lung matrix promotes myofibroblast transdifferentiation. We further demonstrate that the phenotype-driving signal was mediated through specific, central redistribution and activation of endogenous fibroblast NMII. When fibroblasts encounter the stiffer, more densely packed ECM of the fibrotic lung, they develop thick central F-actin stress fibers, which are comprised of phosphorylated NMII and other contractile proteins such as alpha-smooth muscle actin (α-SMA). This leads to the maturation of focal adhesions and the development of fully mature, contractile, matrix-protein-expressing myofibroblasts, which accumulate and perpetuate ongoing scar formation (24). NMII function is regulated at multiple levels. Phosphorylation of both the light and heavy chains regulates activation while ATP-binding to the motor head domain regulates NMII contractile activity. Assembly into filaments free diffusion of NMII throughout the cytoplasm of the cell is regulated by changes in myosin conformation (23). While the role of myosin light chain (MLC) phosphorylation in fibrotic diseases is well-understood, the fibrosis-modulating role of conformational changes in NMII and its redistribution within the cell remain elusive (14, 17, 25, 26). As the calcium-binding protein S100A4 (S100A4) has been shown to bind to NMII heavy chain tails and regulate NMII disassembly, we reasoned that S100A4 could mediate actomyosin complex assembly leading to myofibroblast transdifferentiation and the pro-fibrotic phenotype.

S100A4 is one of 21 known S100 family members that have been implicated in the calcium-dependent regulation of numerous intracellular activities including the assembly and disassembly of cytoskeletal proteins. The study of S100A4 action has broad biological and clinical implications as S100A4 has also been shown to promote fibrosis in the liver, heart, skin, and kidney (27, 28, 29, 30). S100A4 has also been shown to play an important role in cancer cell proliferation, metastasis, and invasiveness (31, 32, 33). Calcium binding to S100A4 induces a conformational change that exposes a large hydrophobic pocket available to bind the NMII-A tail fragment and thereby promote NMII-A filament disassembly, unraveling NMII-A and freeing NMII-A to rapidly diffuse throughout the cytoplasm (34, 35). Based on these observations and our previous work, we investigated whether endogenous fibroblast S100A4 plays a critical role in NMII-A localization to promote myofibroblast transdifferentiation and perpetuate fibrosis.

## Results

### S100A4 localizes to the cytoplasm and is upregulated with increasing ECM stiffness

**Figure 1:**
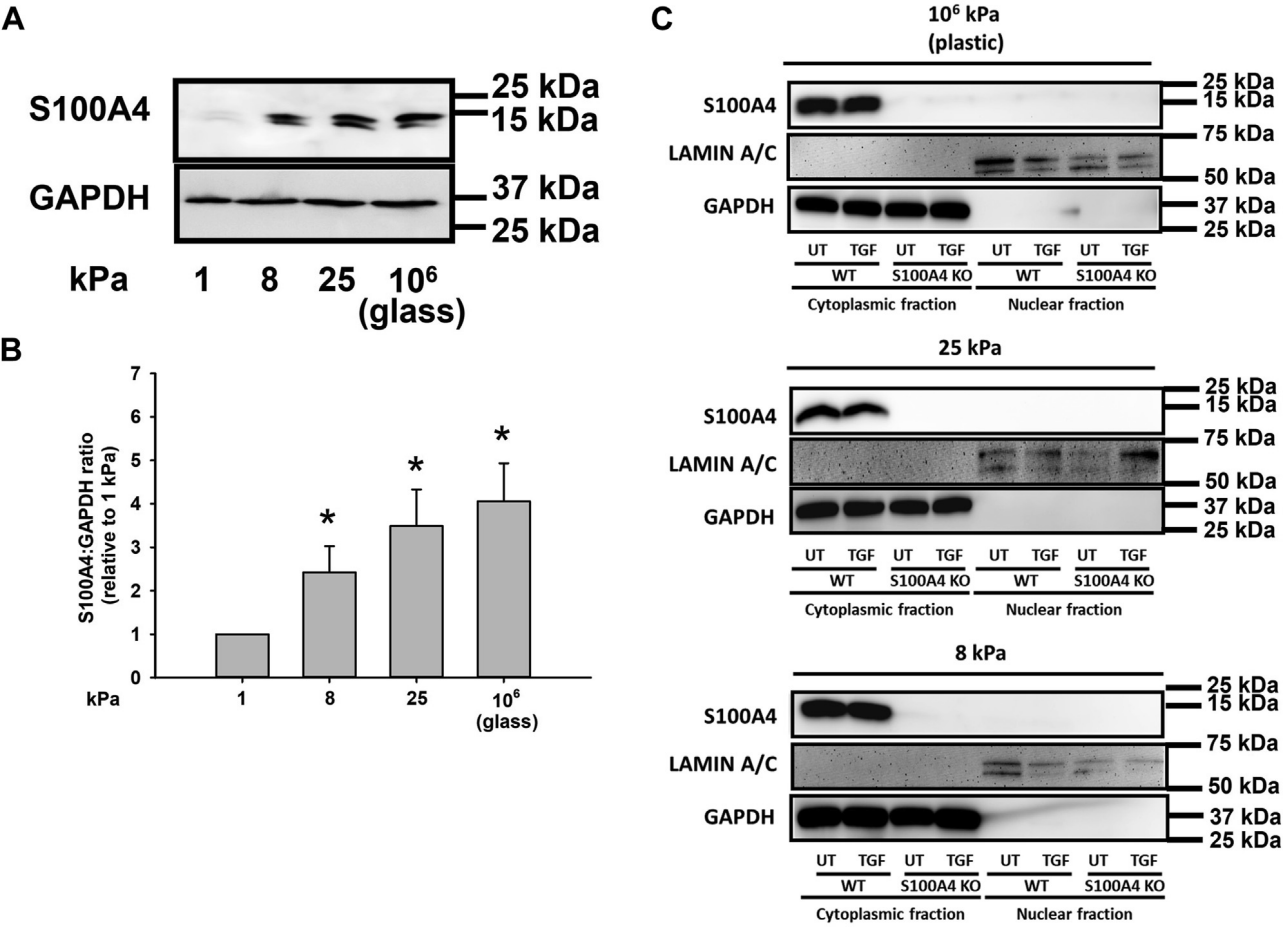
**S100A4 protein localizes to the cytoplasm and is upregulated with increasing ECM stiffness in primary lung fibroblasts.** NL HLFs were plated on fibronectin-coated polyacrylamide gels of indicated stiffnesses, or fibronectin-coated tissue culture-treated plastic. *A*, representative immunoblots of S100A4 and GAPDH from cell lysates. *B*, quantification of S100A4/GAPDH ratio from (*A*). Data represent mean ratio from three independent experiments. ∗denotes *p* < 0.05 compared to 1 kPa by one-way ANOVA. *C*, representative immunoblots from nuclear and cytoplasmic extracts; Lamin A/C was used as a nuclear marker and GAPDH was used as a cytoplasmic marker. There was no detection of nuclear S100A4 with increasing stiffness or addition of TGFꞵ. Each experiment was performed in triplicate.

To determine whether S100A4 expression is sensitive to the biophysical properties of the ECM, we plated normal human lung fibroblasts (NL HLFs) onto fibronectin-coated polyacrylamide gels of stiffnesses in the range of normal lung matrix (1 kPa), fibrotic lung matrix (8–25 kPa), and supra-physiologically stiff tissue culture conditions (10^6^ kPa). With increasing stiffness, protein expression of S100A4 increases up to 4 fold higher than that on normal lung matrix, on fibronectin-coated glass substrates (10^6^ kPa, *p* < 0.05 1 kPa *versus* all other stiffnesses) (Fig. 1, *A* and *B*). As others have demonstrated the role of nuclear S100A4 in lung mesenchymal progenitor cell self-renewal through its effect on p53 proteosomal degradation (36), we measured the subcellular distribution of S100A4 by nuclear/cytoplasmic fractionation. As seen in Figure 1*C*, S100A4 remained in the cytosolic fraction under conditions of both increasing stiffness or following the addition of TGFβ. These data demonstrate that S100A4 expression is mechanosensitive and that S100A4 protein localizes to the cytoplasm under conditions used herein.

### Endogenous fibroblast S100A4 is necessary for cell spreading in response to a pathophysiological range of matrix stiffness

**Figure 2:**
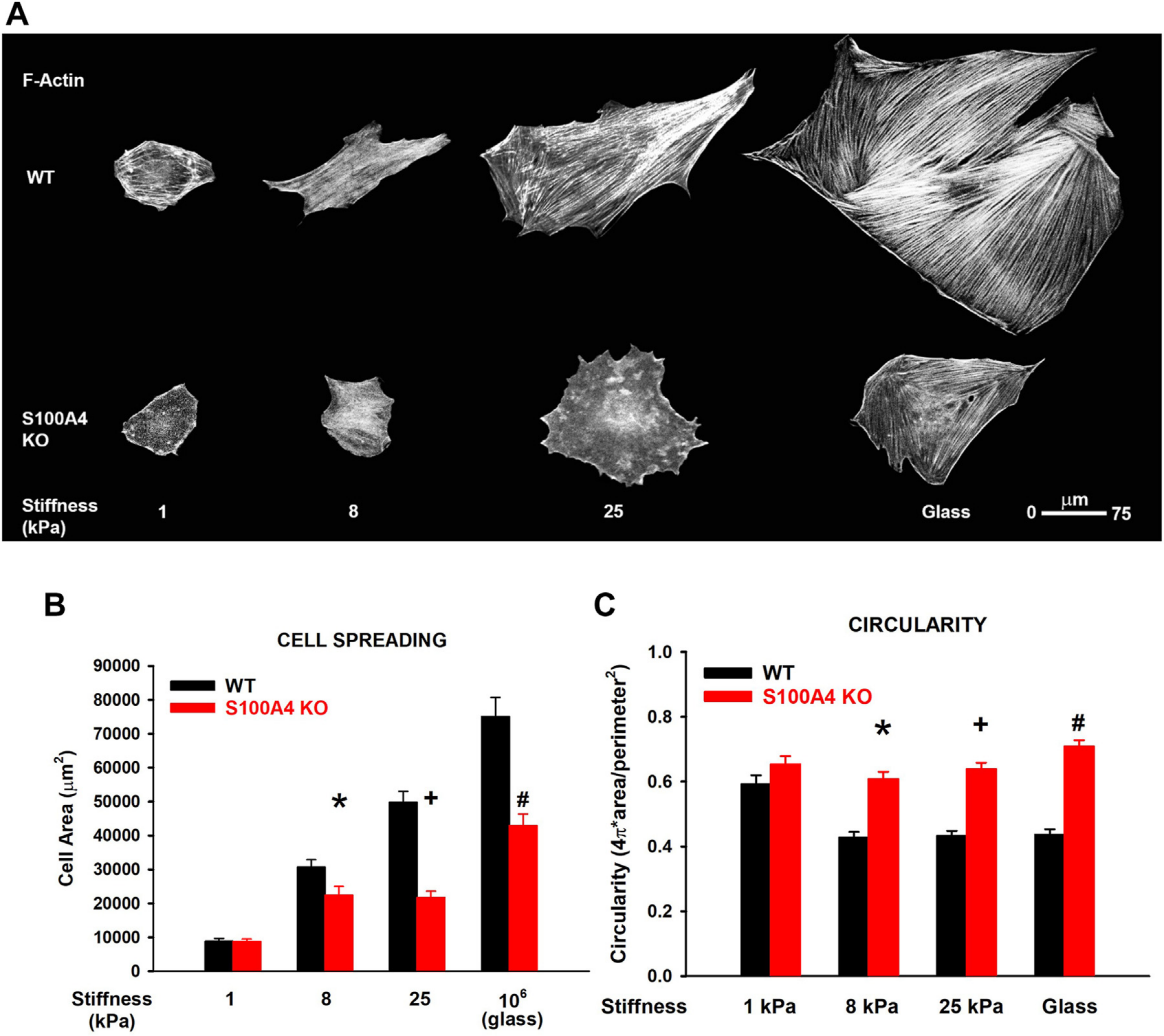
**Endogenous fibroblast S100A4 is necessary for cell spreading in response to pathophysiological range substrate stiffness.** MLFs were plated and allowed to spread (24 h) on fibronectin-coated polyacrylamide gels of indicated stiffnesses or fibronectin-coated glass. *A*, representative photomicrographs (20x/0.4NA orig. mag) of WT *versus* S100A4 KO fibroblasts phalloidin-labeled for F-actin. *B*, quantification of cell area from (*A*). ∗ denotes *p* = 0.02 compared to WT 8 kPa, + denotes *p* < 0.001 compared to WT 25 kPa, # denotes *p* < 0.001 compared to WT glass; two-tailed *t* test. *C*, quantification of cell circularity (4π × area/perimeter^2^) from (*A*). ∗ + # all denote *p* < 0.001 compared to WT for each stiffness; two-tailed *t* test. Data reflect means of six independent experiments, n >10 cells/condition per experiment.

NMII is known to generate the contractile force necessary for cell spreading, and the cortical tension necessary to maintain cell shape (37, 38). We therefore compared the morphology of WT and S100A4 KO mouse lung fibroblasts (MLFs) after plating on varying substrate stiffnesses that ranged from normal (1 kPa), to fibrotic lung (8–25 kPa), and to standard tissue culture conditions (10^6^ kPa). On normal lung substrates (1 kPa), there was no difference in size or shape between WT or S100A4 KO fibroblasts (Fig. 2, *A*–*C*). However, with increasing substrate stiffness in the fibrotic range (8–25 kPa), cell spreading was impaired in the S100A4 KO fibroblasts. For example, at the fibrotic range (25 kPa), there was a 56% reduction in the spreading of S100A4 KO fibroblasts (area = 3825 *versus* 8761 μm^2^, *p* < 0.001 *versus* WT, Fig. 2*A*). Similarly, there was a progressive increase in circularity in S100A4 KO fibroblasts. At 25 kPa, there was a 33% increase in circular shape in S100A4 KO fibroblasts (0.639 *versus* 0.433 A.U., *p* < 0.001 *versus* WT, Fig. 2, *A* and *C*). These data suggest that endogenous fibroblast S100A4 regulates cell spreading and cell shape in response to substrate stiffness.

### S100A4 loss results in an NMII-A-selective dense peripheral ring in response to pathophysiologic-range substrate stiffness

**Figure 3:**
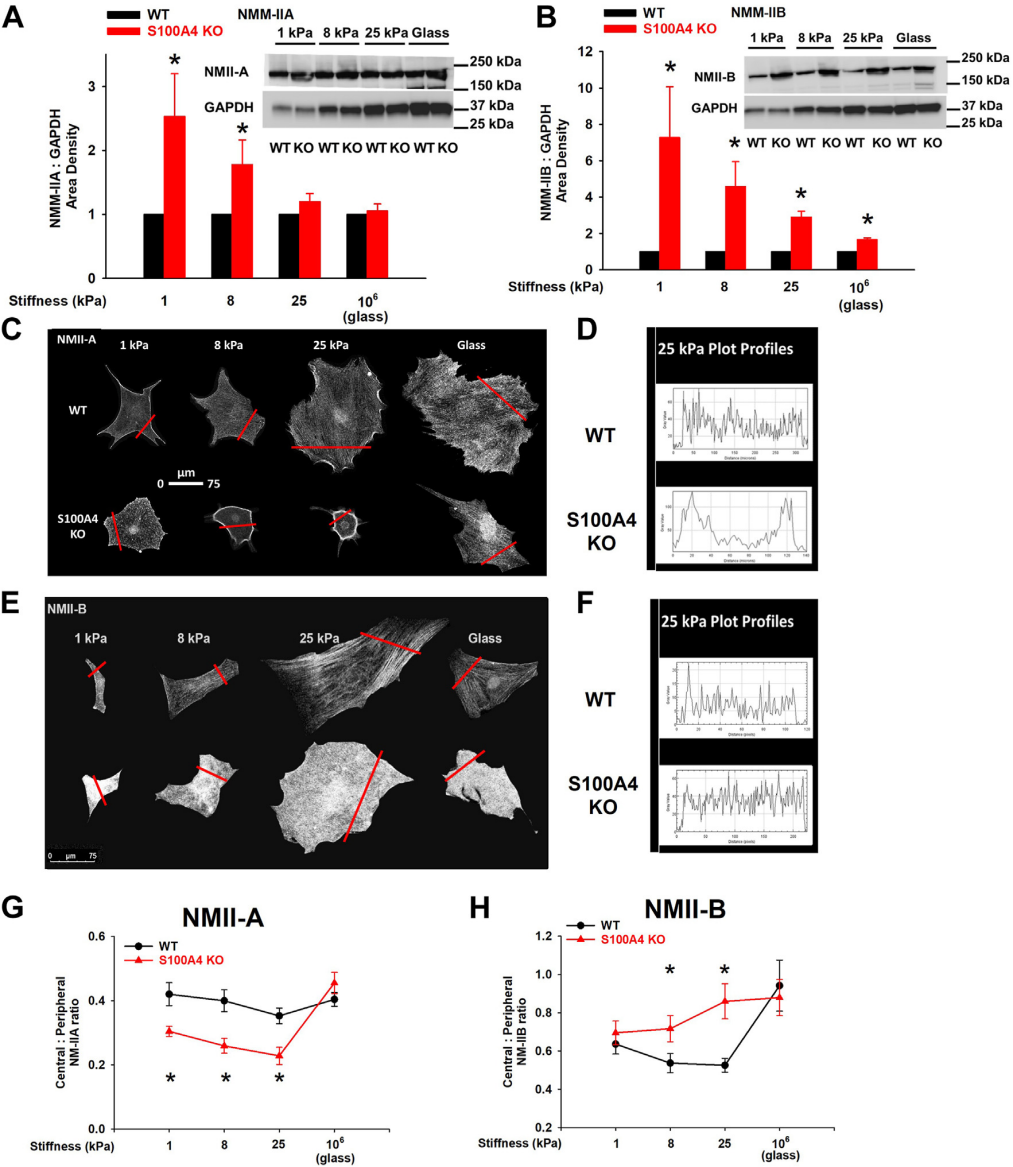
**S100A4 mediates stiffness-dependent dysregulation of both NMII-A and NMII-B expression but selectively drives central accumulation of NMII-A in response to pathophysiologic range substrate stiffness (1–25 kPa).** MLFs were treated as in Figure 2. Representative immunoblots with quantification of area density of (*A*) NMII-A and (*B*) NMII-B relative to GAPDH. Data from three independent experiments.∗denotes *p* < 0.05 compared to WT on corresponding stiffness by *t* test. Representative photomicrographs (20x orig. mag) of WT *versus* S100A4 KO fibroblasts labeled for (*C*) NMII-A or (*E*) NMII-B. *Red lines* indicate where plot profile was taken. Plot profiles of (*D*) NMII-A and (*F*) NMII-B intensity in WT (*top*) *versus* S100A4 KO (*bottom*) fibroblasts on 25 kPa gels as shown in (*C*) and (*E*). Comparison of central to peripheral (*G*) NMII-A and (*H*) NMII-B ratio in WT *versus* S100A4 KO fibroblasts. Data pooled from three experiments, n >10 cells/condition. ∗denotes *p* < 0.05 compared to WT at each stiffness by *t* test.

Given our findings of S100A4-dependent regulation of fibroblast size and shape, and the previous studies that implicate S100A4 in modulating NMII-A filament turnover (34, 35), we hypothesized that S100A4 could modulate the organization and activity of NM-II in fibroblasts. Specifically, we focused on NMII-A and NMII-B, which are the major NMII paralogs expressed in fibroblasts and are known to cooperate during assembly of contractile cytoskeletal structures (39, 40, 41, 42, 43). While there is no significant difference in NMII-A protein levels between WT and S100A4 KO MLFs, there are significantly higher NMII-B expression levels in S100A4 KO fibroblasts on softer substrates that fall to near WT levels with increasing substrate stiffness (Fig. 3, *A* and *B*). Interestingly, the effect of S100A4 loss on the subcellular localization of NMII-A and NMII-B in response to increasing substrate stiffness differs. In the stiffness range of fibrotic lung (8–25 kPa), S100A4 KO fibroblasts demonstrate a localized, intense peripheral ring of NMII-A with a paucity of central NMII-A, as compared to WT (Fig. 3, *C* and *D*). In contrast, S100A4 KO fibroblasts demonstrate no predilection for central or peripheral NMII-B distribution with increasing substrate stiffness (Fig. 3, *E* and *F*). Importantly, the S100A4 distribution-related differences in both NMII-A and NMII-B were limited only to pathophysiologic range substrate stiffnesses that exist under conditions of fibrotic lung disease (8–25 kPa), and the effects of S100A4 loss were lost on standard tissue culture substrate stiffnesses (10^6^ kPa) (Fig. 3, *G* and *H*). Collectively, these findings have broad implications for the *in situ* role of S100A4 in physiologic processes and diseases.

### Intracellular S100A4 is required for myofibroblast transdifferentiation in response to pathophysiological range matrix stiffness

Remodeling of the actomyosin cytoskeleton with the accumulation of thick, central, contractile actomyosin stress fibers containing α-SMA is a defining feature of myofibroblasts (44). In order to determine whether our observations regarding lack of central accumulation of NMII upon S100A4 loss can impair myofibroblastic actin remodeling, we measured the distribution of filamentous, cytoplasmic actin (F-actin) using phalloidin and α-SMA using a specific antibody to α-SMA in WT and S100A4 KO MLFs in response to increasing substrate stiffness and the pro-fibrotic growth factor TGFβ.

**Figure 4:**
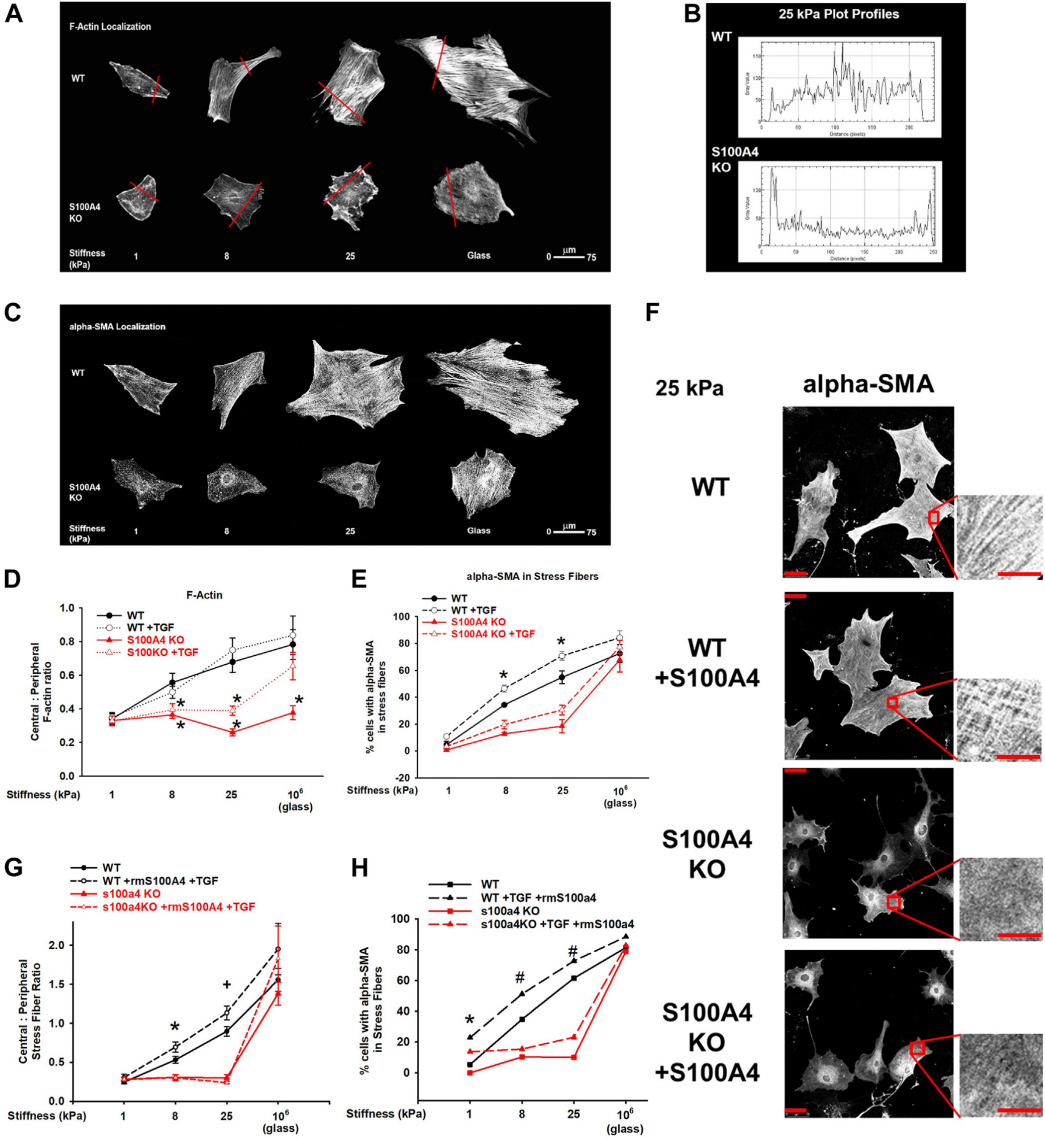
**Intracellular S100A4 is required for myofibroblast transdifferentiation in response to physiological range substrate stiffness and TGFꞵ.** MLFs were plated as in Figure 2 (24 h). *A*, representative photomicrographs (20x/0.4NA orig. mag) of WT *versus* S100A4 KO fibroblasts labeled for F-actin. *Red lines* indicate where plot profile was taken. *B*, plot profile on 25 kPa from (*A*). *C*, representative photomicrographs (20x/0.4NA orig. mag) labeled for α-SMA. *D*, comparison of central to peripheral stress fiber ratio from (*A*). Data pooled from five experiments, n >10 cells/condition. ∗denotes *p* < 0.05 compared to WT at each stiffness by *t* test. *E*, comparison of % cells with α-SMA colocalized to F-actin stress fibers from (*C*). Data pooled from three experiments, n >30 cells/condition. ∗ denotes *p* < 0.05 compared to WT at each stiffness by one-way ANOVA/Dunnett’s test. *F*, representative images (20x/0.4NA orig. mag) ± exogenous recombinant murine S100A4 (1 μg/ml), labeled for α-SMA. Inset shows α-SMA incorporated into stress fibers only in cells with endogenous S100A4 (WT). *Red scale bar* = 75 μm; inset *red scale bar* = 25 μm. *G*, quantification of central to peripheral stress fiber ratio ± exogenous rmS100A4. ∗ denotes *p* < 0.001 compared to S100A4 KO groups by one-way ANOVA/Tukey test. *H*, quantification of the percentage of cells with α-SMA incorporated into stress fibers ± exogenous rmS100A4 and TGFꞵ (2 ng/ml). ∗ denotes *p* = 0.01 compared to other groups on same stiffness, # denotes *p* < 0.001 compared to other groups on same stiffness. Performed in triplicate, n >30 cells/condition per experiment.

On pathophysiologic range substrate stiffnesses, loss of S100A4 resulted in loss of central F-actin stress fibers (62% reduction in central to peripheral stress fiber ratio; Fig. 4, *A*, *B*, and *D*), and a marked reduction of fibroblasts with α-SMA incorporation into stress fibers (of 67%, *p* = 0.006, Fig. 4, *C* and *E*), as compared to WT. However, as we observed above, S100A4 loss had no effect on α-SMA incorporation into central actin stress fibers in fibroblasts on standard tissue culture substrates (Fig. 4, *C* and *E*). Furthermore, the addition of TGFβ failed to compensate for the loss of S100A4 on pathophysiologic substrates (Fig. 4, *A*–*E*). Together, these data demonstrate that S100A4-NMII-A interactions mediate the cytoskeletal remodeling essential for myofibroblast transdifferentiation, a fundamental driver of fibrosis in multiple organs.

**Figure 5:**
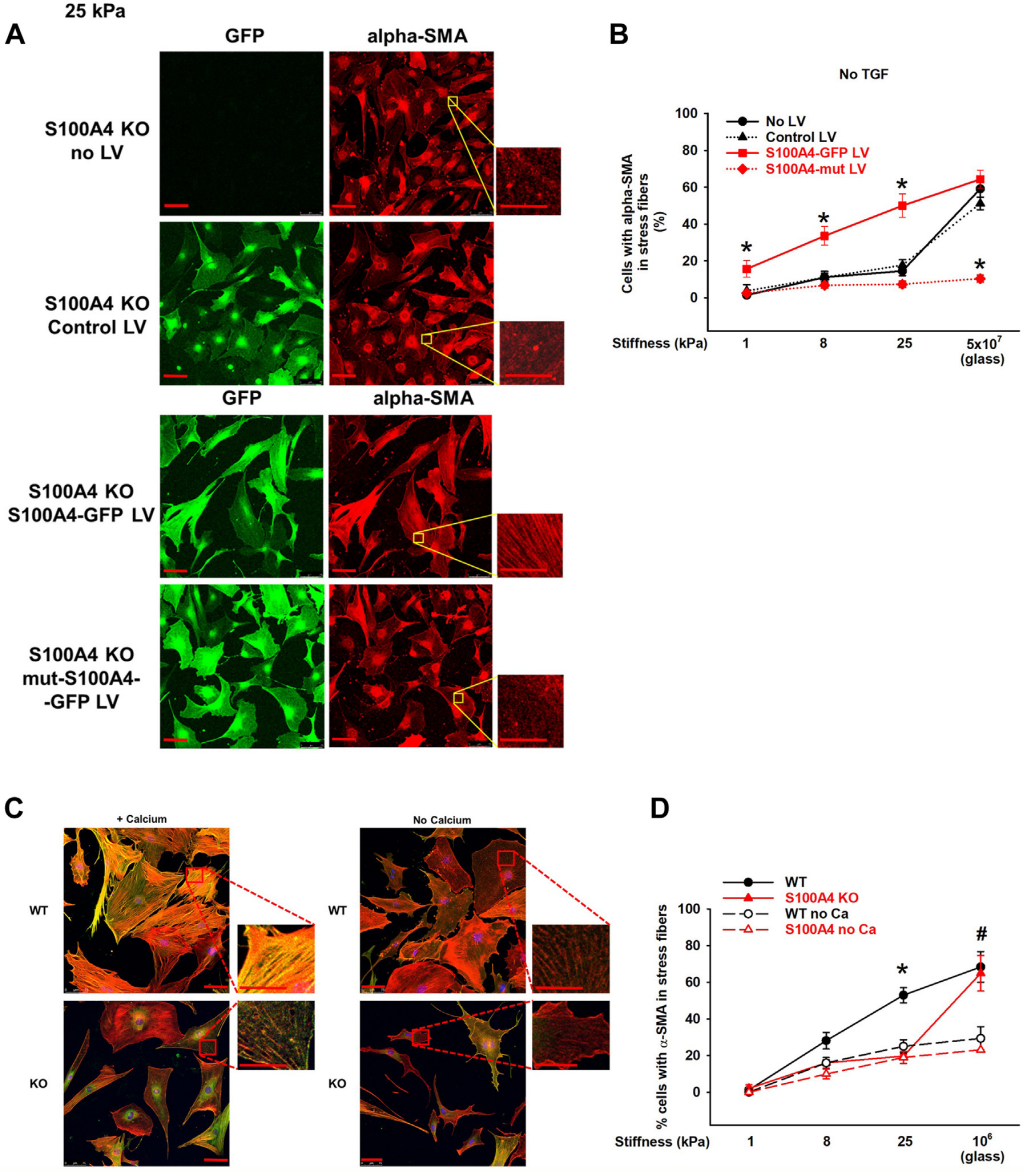
**Endogenous fibroblast S100A4 mediates myofibroblast transdifferentiation in response to physiologic range stiffness in a calcium-dependent manner.** S100A4 KO MLFs were plated on 25 kPa gels following transfection with lentiviral vectors expressing GFP only (Control LV), S100A4 and GFP (S100A4-GFP LV), or a mutant S100A4 that cannot be activated by calcium and GFP (mut-S100A4-GFP LV), or an untransfected control with no lentivirus (No LV). *A*, representative photomicrographs (20x/0.4NA orig. mag.). *Green* represents cells that were successfully transfected. *Red* indicates α-SMA. Inset demonstrates α-SMA incorporated into stress fibers only in the S100A4 KO cells expressing the wild-type S100A4-GFP (third row). *Red scale bar* = 75 μm; *inset red scale bar* = 25 μm. *B*, quantification of the percentage of cells with α-SMA in stress fibers under the conditions of (*A*). Only S100A4 KO cells rescued with LV expressing wild type S100A4 demonstrated stiffness-dependent increase in α-SMA incorporation into stress fibers (*red squares*). ∗ denotes *p* < 0.05 compared to other conditions within same stiffness by one-way ANOVA/Student-Newman-Keuls test. The calcium-inactivatable mutant was unable to form stress fibers on any stiffness (*red diamonds*). ∗ denotes *p* < 0.001 by one-way ANOVA/Student-Newman-Keuls test. *C*, representative photomicrographs (20x/0.4NA orig. mag.) of WT *versus* S100A4 KO fibroblasts on 25 kPa gels in calcium-containing media (no TGFꞵ, *left panel*) or in medium without calcium (no TGFꞵ, *right panel*). Merged image demonstrates α-SMA (*green*), F-actin (*red*), and α-SMA colocalized to stress fibers (*yellow*) only in WT + calcium condition. *Red scale bar* = 75 μm; *inset red scale bar* = 25 μm. *D*, quantification of the percentage of cells with α-SMA colocalized to stress fibers in WT *versus* S100A4 KO fibroblasts on varying stiffness substrates ± extracellular calcium (no TGFꞵ added). ∗ denotes *p* = 0.005 WT vs all other groups; # denotes WT *p* = 0.022 vs all “no calcium” groups (ANOVA/Dunnett’s test).

Previous work has demonstrated that exogenously added recombinant murine (rm) S100A4 can induce α-SMA and collagen expression in an immortalized lung cell line and can induce proliferation, migration, and expression of α-SMA and collagen I in primary MLFs (45, 46). We first confirmed this prior work showing exogenous rmS100A4 can induce cytoskeletal remodeling in both WT and S100A4 KO MLFs plated on standard tissue culture conditions (10^6^ kPa; Fig. 4, *G* and *H* - glass). In contrast, the cytoskeletal remodeling of myofibroblast transdifferentiation in fibroblasts plated on pathophysiologic range substrates specifically required intracellular S100A4 (Fig. 4, *F*–*H*), as exogenous rmS100A4 failed to rescue the defective myofibroblast phenotype in S100A4 KO fibroblasts. Exogenous rmS100A4 specifically did not induce central stress fiber formation (Fig. 4*F* bottom panel; Fig. 4*G*, red lines), or incorporation of α-SMA into stress fibers, even with the addition of TGFβ (Fig. 4*H*, red lines). Furthermore, exogenously added rmS100A4 had no effect on cytoskeletal remodeling in fibroblasts plated on pathophysiologic range substrates, whether or not they express S100A4 intracellularly (Fig. 4, *F*–*H*). In summary, our observations demonstrate that endogenous fibroblast S100A4 plays a dominant role in determining cytoskeletal remodeling under pathophysiologic conditions. To further substantiate the requirement for intracellular S100A4 in determining the myofibroblast phenotype, we performed gain-of-function experiments for S100A4 using lentiviral expression of a S100A4-GFP fusion protein (S100A4-GFP LV). These data demonstrate a clear rescue of the myofibroblast phenotype in MLFs that lack S100A4 plated on substrates in the pathophysiologic range (Fig. 5, *A* and *B*). Taken together, the results of these gain-of-function experiments suggest that in the environment of a stiffened fibrotic lung, intracellular, but not secreted, S100A4 is the dominant driver of myofibroblast differentiation.

### S100A4 mediates myofibroblast transdifferentiation in response to pathophysiologic range stiffness in a calcium-dependent manner

We and others have previously shown that extracellular calcium is required for transdifferentiation of fibroblasts to myofibroblasts under standard *in vitro* tissue culture conditions (14, 47). One prominent driver of myofibroblast transdifferentiation is the stiffness-dependent sensitization of a membrane cation channel, transient receptor potential vanilloid-4 (TRPV4) through its effect on calcium influx (14, 48, 49). As calcium induces a conformational rearrangement in S100A4 required for its binding to NMII-A, we hypothesized that S100A4-dependent myofibroblast transdifferentiation will be calcium-dependent. When expressed in S100A4 KO fibroblasts, an S100A4 mutant that is defective in calcium-binding (mut-S100A4-GFP LV) is incapable of forming stress fibers or myofibroblasts (Fig. 5*A*, bottom row, Fig. 5*B*, red diamonds). Moreover, neither WT nor S100A4 KO fibroblasts can transition to myofibroblasts in response to any substrate stiffness, in the absence of extracellular calcium (Fig. 5*C*). There was a 50% to 60% reduction in α-SMA incorporation into stress fibers in the absence of calcium in both WT and S100A4 KO fibroblasts on 25 kPa substrates (Fig. 5, *C* and *D*, dotted lines). As before, supra-physiologic substrate stiffness allowed fibroblasts to overcome the lack of S100A4 (Fig. 5*D*, filled red triangles). Together these data imply that the influx of extracellular calcium and its activation of fibroblast S100A4 is necessary for myofibroblast transdifferentiation in the mechanical environment of fibrotic lung, thereby perpetuating the pro-fibrotic phenotype.

### Loss of S100A4 selectively locks peripheral NMII-A fibers in their filamentous conformation

**Figure 6:**
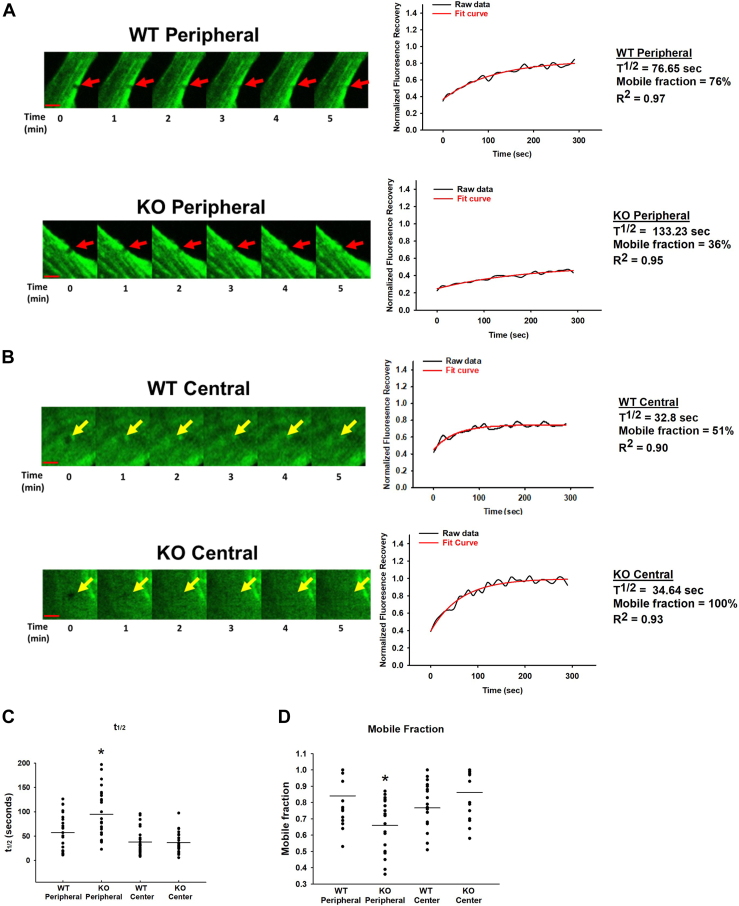
**Loss of S100A4 selectively locks peripheral NMII-A fibers in their filamentous conformation.** WT and S100A4 KO MLFs were plated onto fibronectin-coated glass coverslip-*bottom dishes*, transduced with a plasmid expressing a NMII-A-EGFP fusion protein, and 2 μm regions of interest were bleached in the periphery and center of each cell. Representative kymographs (63x/1.32NA orig. mag.) of NMII-A-EGFP (*A*) peripheral *versus* (*B*) central regions of WT vs S100A4 KO fibroblasts demonstrating recovery of bleached regions (*red arrows* represent peripheral regions and *yellow arrows* represent central regions), accompanied by fluorescence recovery curves. *Red scale bar* = 50 μm. Comparison of (*C*) peripheral *versus* central half time of recovery (t_1/2_, ∗denotes *p* = 0.004 compared to WT peripheral) and (*D*) mobile fraction (∗denotes *p* < 0.001 compared to WT peripheral). Data represents total of five separate FRAP experiments, n >20 cells for each condition.

To understand the underlying mechanism whereby S100A4 loss induces thick peripheral NMII-A rings, we performed fluorescence recovery after photobleaching (FRAP) on WT and S100A4 KO MLFs transfected with a plasmid containing an NMII-A-EGFP fusion protein. Bleaching and recovery were analyzed in the peripheral and central locations of each cell (Supplemental Video 1). NMII-A filaments have a markedly slower recovery after photobleaching selectively in the cell periphery of S100A4 KO fibroblasts, as compared to that of WT (t_1/2_ 40% longer, *p* = 0.004, Fig. 6, *A*–*C*). These data can be explained by either a decrease in diffusion and/or an increase in the assembly of NMII-A selectively in the periphery of the S100A4 KO fibroblasts. Furthermore, the mobile fraction, as measured by the extent of recovery after photobleaching, of NMII-A is reduced by 21% in the periphery of S100A4 KO fibroblasts, as compared to WT (*p* < 0.001, Fig. 6*D*). The reduced recovery can be explained by a limitation in the available pool of NMII-A monomers trapped in the periphery of S100A4 KO fibroblasts. Collectively, these FRAP data suggest that peripheral NMII-A fibers in S100A4 KO fibroblasts are unable to disassemble, and thereby become locked in a filamentous state selectively in the periphery of these cells.

### Loss of S100A4 selectively locks peripheral actin in filamentous bundles

**Figure 7:**
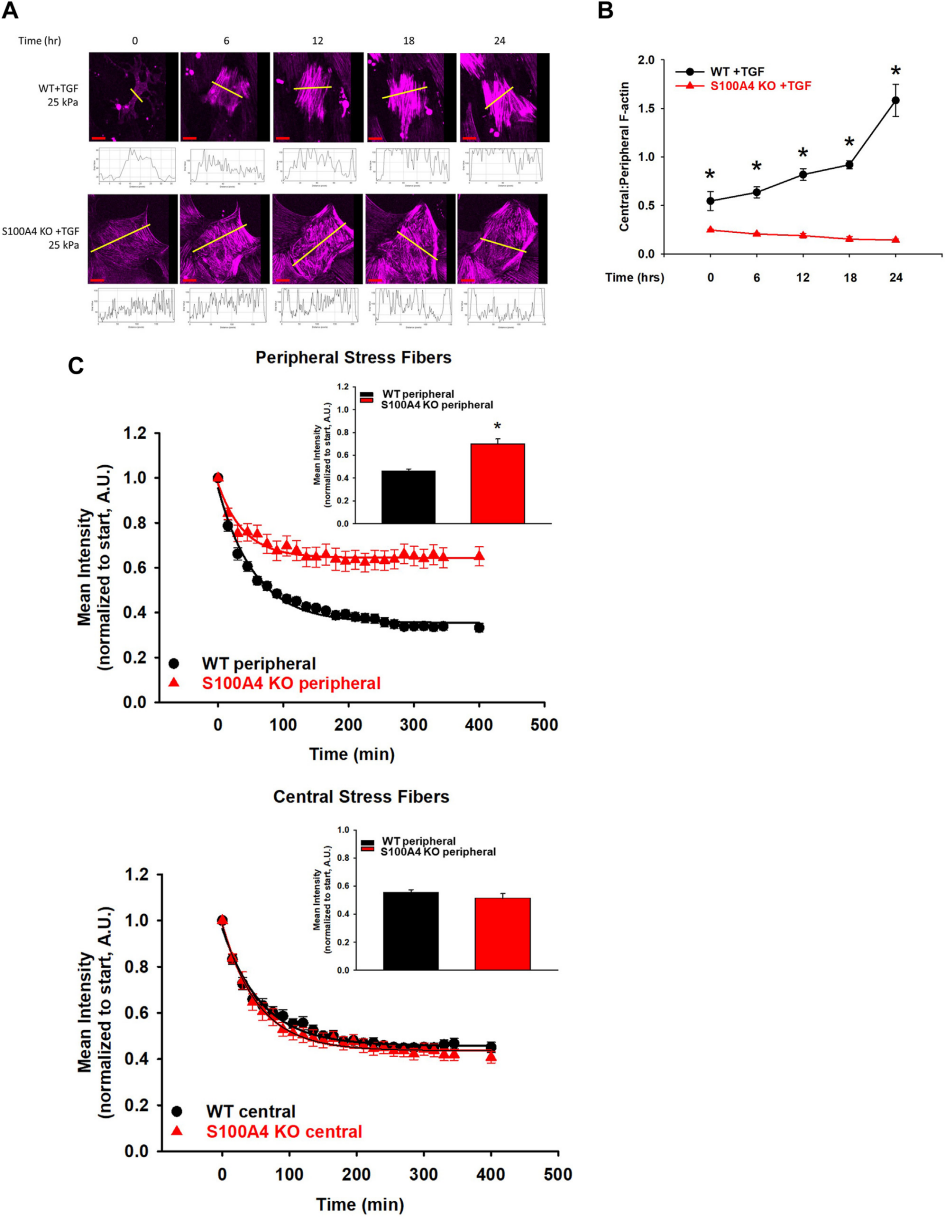
**Loss of S100A4 selectively locks peripheral actin in filamentous bundles.** WT or S100A4 KO MLFs were plated on 25 kPa fibronectin-coated gels in the presence of SiR-actin (75 nM) and TGFβ (2 ng/ml, 24 h). *A*, representative photomicrographs (20x orig. mag.) of time-lapse microscopy. *Yellow lines* indicate the location of the mean fluorescence intensity measurements as reflected in the plot profile below the photomicrograph. *Red scale bar* = 75 μm. *B*, quantification of central:peripheral F-actin stress fiber ratio over time from (*A*). ∗ denotes *p* < 0.05 of S100A4KO + TGFβ compared to WT +TGFβ at the indicated time points using *t* test. *C*, WT or S100A4 KO fibroblasts were plated as before in the presence of SiR-actin for 4 h, which was then washed off, then incubated in TGFβ (2 ng/ml, 6 h). Mean fluorescence intensity was measured over time in peripheral (*left panel*) and central (*right panel*) regions. Inset shows mean fluorescence intensity at 105 min ∗ denotes *p* < 0.001 compared to WT by *t* test. All experiments were performed in triplicate, n >30 cells/condition per experiment.

We analyzed actin dynamics in WT *versus* S100A4 KO MLFs on fibrotic lung matrix-range stiffness (25 kPa) after stimulation with TGFβ utilizing the fluorogenic live-cell F-actin specific probe, SiR-actin (SA). Increases in fluorescence labeling intensity with SA denote actin filament stability in live cells (50). Loss of S100A4 changed the distribution of stable actin filaments from central actin stress fibers in WT cells into actin bundles in the cell periphery, as measured by continuous SA labeling. (Fig. 7*A* and Supplemental Video 1a). Central:peripheral stress fiber ratio in WT fibroblasts is 1.5 fold higher than that of S100A4 KO MLFs (*p* < 0.001) (Fig. 7*B*). Furthermore, the slowed decay of the fluorescent signal selectively in the periphery of S100A4 KO MLFs using SA pulse labeling, documents greater stability of stress fibers in the periphery in these cells (Fig. 7*C* and Supplemental Video 1b). In the absence of S100A4, NMII-A and its binding partner F-actin, remained locked in a peripheral distribution. When taken together with the loss-of-function FRAP results, these data suggest that S100A4 functions to mediate the disassembly of peripheral NMII-A filaments, allowing NMII-A monomers to freely diffuse into the cell center, thereby initiating the development of central actomyosin stress fibers and a functional myofibroblast.

### Loss of S100A4 inhibits focal adhesion maturation and traction force generation in response to pathophysiologic range stiffness

**Figure 8:**
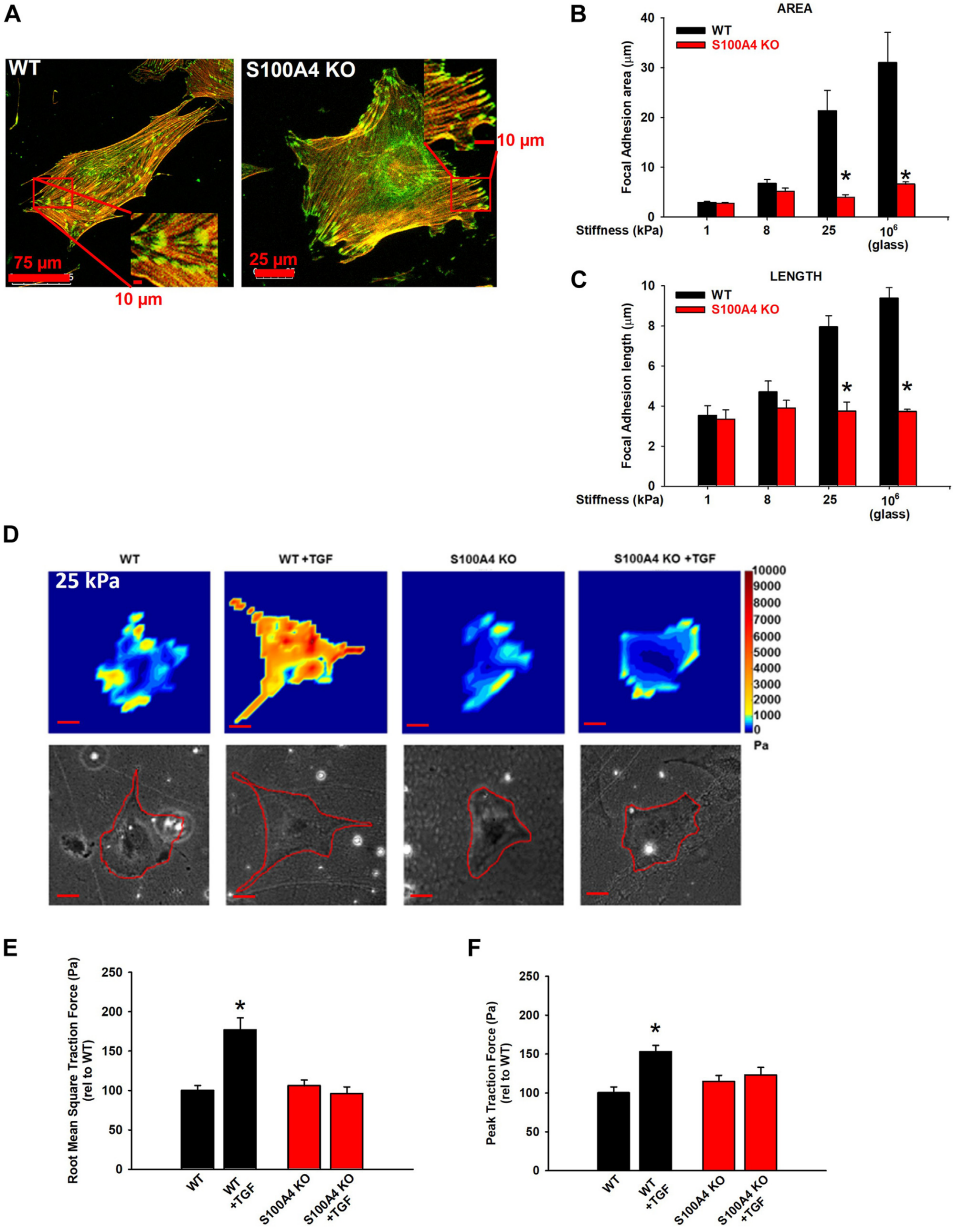
**Loss of S100A4 inhibits focal adhesion maturation and traction force in response to pathophysiologic range stiffness.** WT vs S100A4 KO MLFs were plated on 25 kPa gels overnight in 1% SCM, followed by TGFβ (2 ng/ml, 24 h), and labeled for vinculin (*green*) and F-actin (*red*). *A*, representative photomicrographs (20x orig. mag.) and insets with *yellow* indicating vinculin-containing focal adhesions. Scale bar length as indicated in figure. Quantification of focal adhesion (*B*) area and (*C*) length. ∗ denotes *p* < 0.05 compared to WT at same stiffness by *t* test. Performed in triplicate, n >30 cells analyzed per condition. *D*, for traction force experiments, WT *versus* S100A4 KO MLFs were plated on 25 kPa gels for 1 h ± TGFβ1 (10 ng/ml, 24 h). Representative traction force microscopy heat maps and corresponding phase images (10x orig. mag.). *Red scale bar* = 75 μm. Quantification of (*E*) root mean square traction force and (*F*) peak traction force from (*D*). ∗ denotes *p* < 0.05 compared to WT (ANOVA/Dunnett’s test). Experiments performed in triplicate, n >30 cells analyzed per condition.

It has been well described that fibroblasts, and other cell types, respond to increasing substrate stiffness by matching their internal contractile force with the extracellular forces (51, 52). Mechanistically, this occurs as a result of increased contractility of central stress fibers directed toward focal adhesions, which grow, mature, and thereby strengthen adherence and force transmission to the surrounding matrix substrate. Since our data suggest a key role for S100A4 in regulating stress fiber assembly, we next sought to investigate if S100A4 affects the growth of focal adhesions. Confocal microscopy of dual-labeled (F-actin, vinculin), TGFꞵ-activated WT, and S100A4 KO MLFs shows that loss of S100A4 markedly impairs the normal focal adhesion growth response to increasing substrate stiffness (Fig. 8, *A*–*C*). Concordantly, loss of S100A4 abrogates the mean (by 46%, *p* < 0.001) and peak traction force (by 20%, *p* < 0.001) exerted by S100A4 KO MLFs in response to both pathophysiologic range (25 kPa) mechanical stiffness and to TGFβ (Fig. 8, *D*–*F*). Together, these data demonstrate that S100A4 mediates the functional response to increasing substrate stiffness by growing focal adhesions and by increasing cell contractility, hallmarks of the myofibroblast phenotype.

### S100A4 KO mice are protected from the pro-fibrotic effects of bleomycin

**Figure 9:**
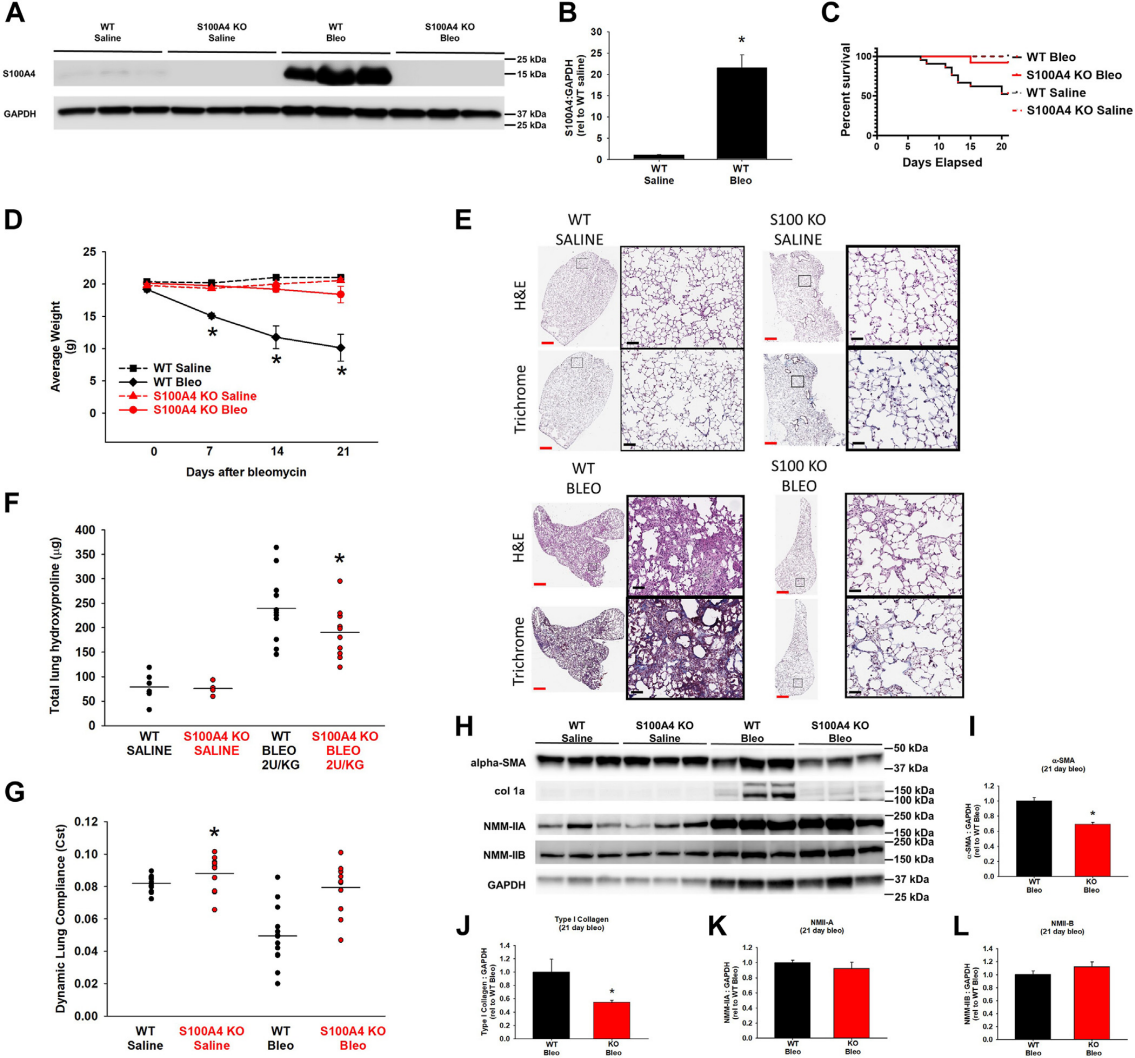
**S100A4 KO mice are protected from the pro-fibrotic effects of bleomycin.** WT or S100A4 KO mice were given oropharangeal bleomycin (2 U/kg) or saline and lungs were harvested at 21 days. *A*, representative S100A4 immunoblots of whole lung lysates. *B*, quantification of S100A4 to GAPDH area density in WT mice from (*A*). ∗denotes *p* < 0.001 compared to WT saline. *C*, survival curves of mouse groups (∗ denotes *p* < 0.001 by Log-rank (Mantel-Cox) test). *D*, comparison of weight loss between mice. ∗ denotes *p* < 0.001 compared to all other groups at each time point after intervention by one-way ANOVA/Tukey test. *E*, representative photomicrographs of trichrome-stained lung tissue (*left* – whole lobe; *right* – 40x orig. mag.). *Red scale bar* = 1 mm, *black scale bar* = 1 μm. *F*, comparison of whole lung hydroxyproline between mouse groups. ∗denotes *p* = 0.03 compared to WT bleomycin 2 U/kg by ANOVA/Student-Newman-Keuls. *G*, comparison of dynamic lung compliance between mouse groups. ∗denotes *p* < 0.0001 compared to change between S100A4 KO groups by *t* test. *H*, representative immunoblots of whole lung lysates for indicated proteins. *I*–*L*, Quantification of (*I*) α-SMA, (*J*) Type I Collagen, (*K*) NMII-A, and (*L*) NMII-B relative to GAPDH from (*H*). Data represents lysates from five mice/group. ∗ denotes *p* < 0.05 compared to WT Bleo.

To investigate the role of S100A4 on pulmonary fibrogenesis *in vivo*, the effect of intra-pulmonary bleomycin was studied in WT and S100A4 KO mice. As expected, S100A4 KO mice did not express S100A4 at baseline or in response to bleomycin (Fig. 9*A*). The WT mice given saline had barely detectable S100A4 expression in the lung, but WT mice demonstrated a greater than 20-fold increase (*p* = 0.003) in S100A4 expression during the fibrotic phase of bleomycin injury (day 21) (Fig. 9, *A* and *B*). The S100A4 KO mice had decreased mortality (*p* < 0.001) (Fig. 9*C*), and a more stable body weight (85% reduction in body weight loss (*p* < 0.001)) (Fig. 9*D*) in response to bleomycin, as compared with WT mice. Importantly, the S100A4 KO mice were significantly protected from bleomycin-induced lung fibrosis. S100A4 KO mice had less lung collagen accumulation after bleomycin (2 U/kg bleomycin, day 21) by trichrome staining (Fig. 9*E*), and as quantitated by measuring the change in hydroxyproline levels (30% less *versus* saline, *p* = 0.03) (Fig. 9*F*), or by measuring type 1 collagen in lung tissue extracts by immunoblotting (Fig. 9, *H* and *J*). S100A4 KO mice had 74% less impaired lung compliance after bleomycin instillation, as compared to WT mice (*p* < 0.001) (Fig. 9*G*). Additionally, the lungs of bleomycin-treated S100A4 KO mice expressed less α-SMA (Fig. 9, *H* and *I*), but there were no differences in the expression of either NMII-A or NMII-B in whole lung lysates, compared to WT mice (Fig. 9, *H*, *K*, and *L*). Collectively, these data demonstrate that S100A4 KO mice are protected from the pro-fibrotic effects of bleomycin, despite having equivalent levels of NMII-A and NMII-B proteins as their bleomycin-instilled WT controls.

### S100A4 is upregulated in IPF fibroblasts and mediates myofibroblast transdifferentiation of MLFs on stiff, fibrotic lung tissue

**Figure 10:**
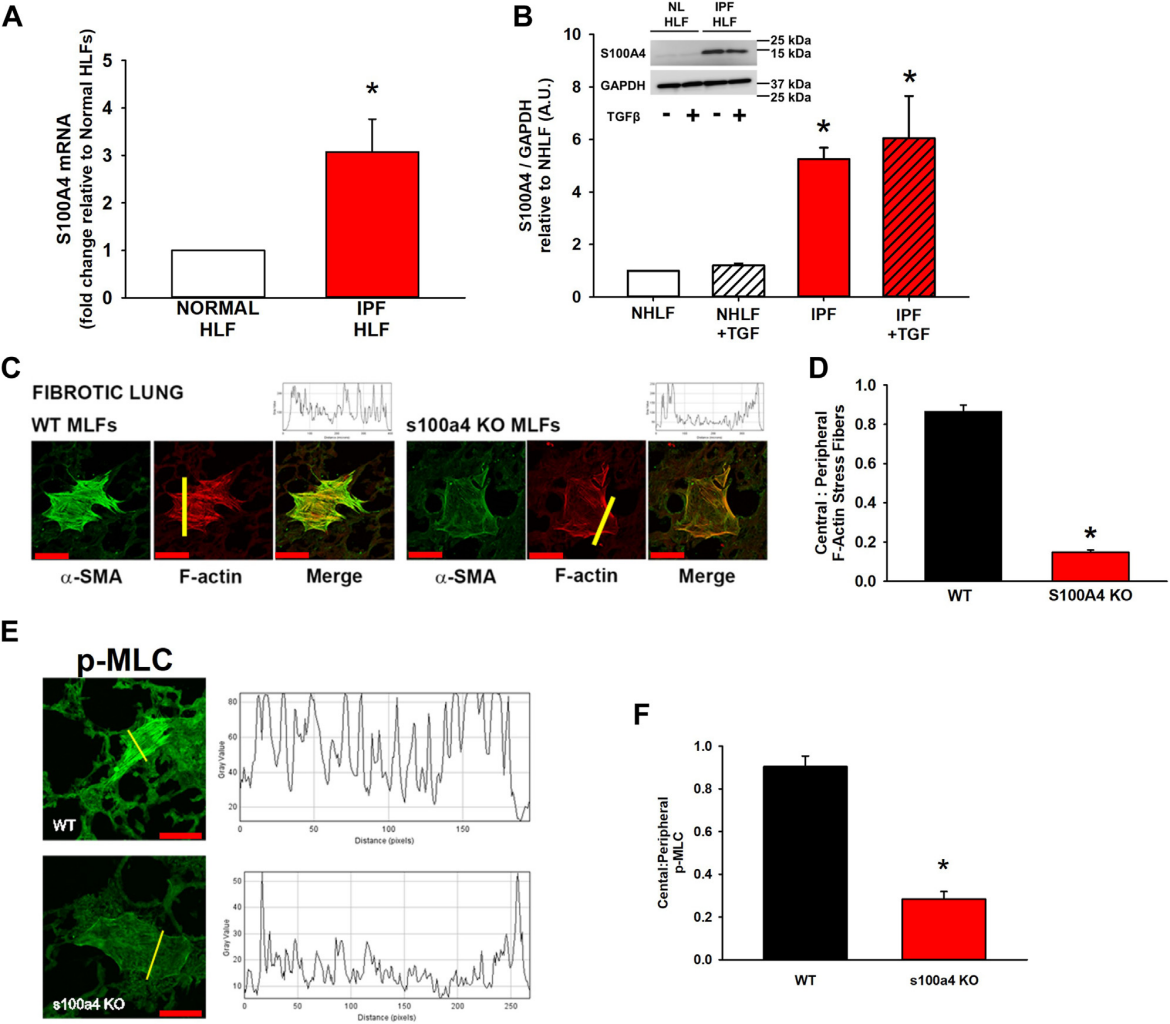
**S100A4 is upregulated in IPF fibroblasts and mediates myofibroblast transdifferentiation of MLFs on stiff, fibrotic lung tissue.***A*, quantification of S100A4 mRNA from normal (NL HLF) and IPF-patient derived fibroblasts (IPF HLF) after plating on fibronectin-coated tissue culture plastic for 24 h. Results derived from mean + SD of three independent patient-derived HLFs from each group, (normalized to GAPDH control, fold change relative to NL HLF mRNA shown, ∗ denotes *p* < 0.001 compared to NL HLF). *B*, representative immunoblots and quantification of S100A4 protein relative to GAPDH from NL HLFs and IPF HLFs ±TGFβ1 (2 ng/ml, 24 h). Data shown are mean + SE from three independent patient samples per group relative to NL HLFs, ∗ denotes *p* < 0.001 compared to the corresponding NL HLF group. *C*, WT and S100A4 MLF were seeded for 24 h onto 10 μm sections of lungs from bleomycin-treated mice (21 days) and labeled for α-SMA (*green*) and F-actin (*red*). Representative confocal photomicrographs (20x/0.4NA orig. mag.) of fibroblast on a lung tissue section. *Red scale bar* = 75 μm. *Yellow bars* indicate from where intensity plot profiles were obtained (above merged image). S100A4 MLF do not form central stress fibers containing α-SMA. *D*, quantification of central:peripheral F-actin ratio from ©. ∗ denotes *p* < 0.001 compared to WT. The experiment was performed in triplicate, n >30 cells were analyzed per group. *E*, representative confocal photomicrographs (20x/0.4NA orig. mag.) of WT *versus* S100A4 KO seeded onto fibrotic lung and labeled for phosphorylated myosin light chain (p-MLC, *green*), with intensity plot profiles generated as in (*D*). *Red scale bar* = 75 μm. *F*, quantification of central:peripheral p-MLC from (*D*). ∗ denotes *p* < 0.001 compared to WT. Three independent experiments were performed, n >30 cells analyzed per group.

**Figure 11:**
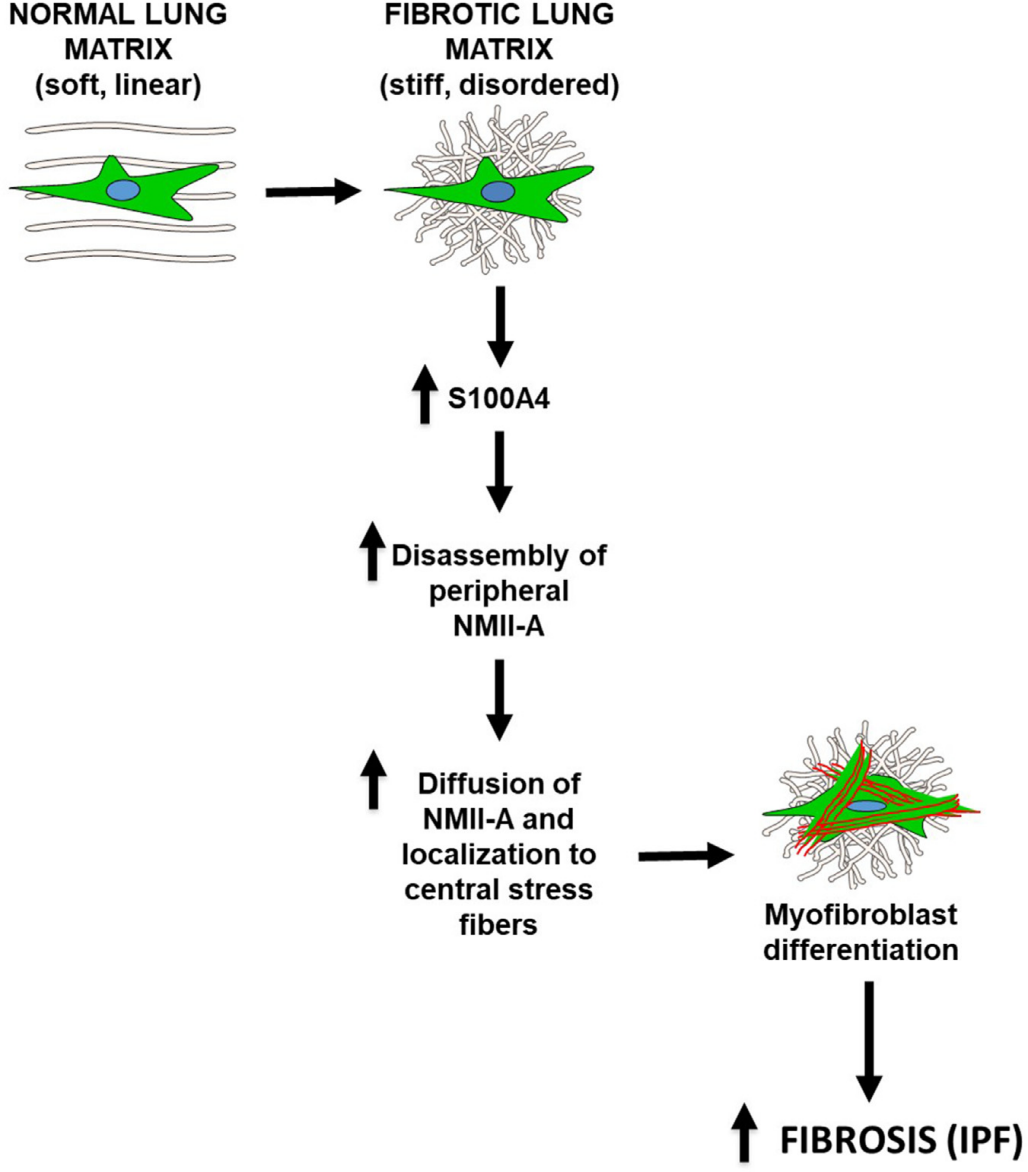
**Model of the role of endogenous fibroblast S100A4 in myofibroblast transdifferentiation and IPF.** As fibroblasts encounter stiff, disordered fibrotic lung matrix, S100A4 is upregulated, the increased S100A4 is locally activated *via* extracellular calcium influx through mechanosensitive ion channels, resulting in disassembly of peripheral NMII-A filaments into their 10S mobile conformation. This conformation allows NMII-A to diffuse to the center of the cell and co-assemble with F-actin in stress fibers to form a fully differentiated, contractile myofibroblast. Accumulation of myofibroblasts in mechanically stiff areas of ongoing fibrosis perpetuate the feed-forward cycle of fibrosis in IPF.

To confirm the relevance of fibroblast-derived S100A4 in human fibrotic lung disease, we demonstrate that S100A4 is upregulated in IPF-patient-derived fibroblasts, both at the mRNA (increased 3-fold, *p* < 0.001) and protein levels (>5-fold, *p* < 0.001) (Fig. 10, *A* and *B*). We have previously demonstrated that lung fibroblasts seeded onto actual fibrotic lung tissue develop into fully differentiated myofibroblasts (14, 17). The paucity of central α-SMA or central F-actin stress fibers in S100A4 KO fibroblasts we observed *in vitro* on homogenous substrates of fibrotic-range stiffness (Fig. 4) is recapitulated in our *ex vivo* lung explant system (Fig. 10, *C* and *D*). S100A4 KO fibroblasts seeded onto fibrotic lung tissue also exhibit decreased activation of myosin in the center of the cell, as measured by central:peripheral ratio of phosphorylated MLC (Fig. 10, *E* and *F*). These data demonstrate that fibroblasts lacking S100A4 lack key characteristics of a fully differentiated myofibroblast when adherent to an actual fibrotic lung matrix. In summary, S100A4 is critical in NMII-A cytoskeletal remodeling of IPF fibroblasts and contributes to the aberrant fibroblast response to the biophysical cues from the fibrotic lung matrix (Fig. 11).

## Discussion

The key finding of this study is that endogenous fibroblast S100A4 is a calcium-dependent, mechanoeffector protein that mediates myofibroblast transdifferentiation through its enhancing effect on peripheral NMII-A fiber disassembly and subsequent central relocation of NMII-A within the cell. This finding was substantiated by several lines of evidence as follows. S100A4-mediated effects are uniquely sensitive to matrix stiffnesses in the pathophysiologic range (8–25 kPa) of fibrotic lung but are lost on supra-physiologically stiff substrates such as standard tissue culture plastic or glass. Endogenous, cytoplasmic, fibroblast S100A4 specifically mediates unraveling of peripheral cytoskeletal proteins NMII-A and F-actin, leading to their redistribution to a central cellular location. This actomyosin redistribution mediates the development of central stress fibers, force-dependent maturation of focal adhesions, and the generation of a contractile myofibroblast. S100A4 loss protects from experimental *in vivo* pulmonary fibrosis, and S100A4 expression is dysregulated in human IPF fibroblasts. In summary, our work reveals a novel pro-fibrotic mechanoeffector function of endogenous fibroblast S100A4-induced cytoskeletal redistribution specifically in response to pathophysiologic range lung matrix stiffnesses.

### Mechanoeffector pathways

Cells respond to numerous types of mechanical stimuli including external pressure, substrate stiffness, flow-dependent stimuli (shear), or osmotic stress (swelling/shrinking) by converting the mechanical stimuli into a biochemical response. As such, mechanotransduction comprises sensing of the mechanical stimulus, the intracellular transduction of a mechanical stimulus, and an effector response to the intracellular signal (53, 54). In fibrotic disorders such as IPF, it has been well described that fibrotic lung tissue is 10-fold stiffer than normal, which in turn activates fibroblasts, resulting in a feed-forward progressive cycle of fibrosis leading to inexorable decline in lung function and respiratory failure (12, 13, 14, 17, 55, 56, 57). In this study, S100A4 is implicated for the first time as a mechanoeffector protein, through its capacity to interact with NMII-A. Several specific findings support this conclusion: (1) the redistribution of NMII-A in response to pathophysiologic range stiffness is lost upon knockout of S100A4, (2) the redistribution of NMII-A is rescued upon re-expression of S100A4 in KO cells, and (3) the FRAP and SiR actin dynamic analyses demonstrate selective retention of a cortical ring of NMII-A and actin in the S100A4 KO fibroblasts, all selectively in response to pathophysiologic range stiffness.

As a first step for mechanotransduction, biophysical forces must be sensed, where our group and others have identified a number of mechanosensitive receptors, such as transient receptor potential vanilloid 4 (TRPV4), a key receptor through which substrate stiffness results in intracellular influx of calcium (14). This receptor and others induce signaling changes in a number of downstream proteins that effect cell phenotypic responses (26, 55, 58, 59, 60, 61). The importance of extracellular calcium influx in mechanotransduction-induced myofibroblast differentiation is supported by our findings of the requirement for extracellular calcium on all ranges of substrate stiffness, as well as the failure of the S100A4 calcium binding-deficient mutant to mediate myofibroblast transdifferentiation in response to pathophysiological range matrix stiffnesses. These data further suggest that S100A4 is the dominant calcium-responsive protein under conditions of pathophysiological range matrix stiffnesses, while other calcium-dependent proteins dominate the response to supra-physiologic range stiffness. While the prominent role of calcium-dependent, cytoplasmic S100A4 binding with NMII-A has been well studied in cell motility and invasion, its role in myofibroblast transdifferentiation has heretofore not been revealed (33, 62). Taken collectively, this novel work implicates S100A4 as a calcium-dependent mechanoeffector protein that participates in the transduction of substrate stiffness into a signal that changes the phenotype of the fibroblast to one that is pro-fibrotic, in the precise range of fibrotic lung matrix.

### Cytoplasmic S100A4 is a key regulator of pathophysiological range stiffness sensitivity *via* NMII-A interaction

Biochemical and cellular studies collectively show that S100A4 can interact with NMII-A in the cytoplasm, and we have previously shown that the subcellular localization of NMII depends on lung tissue matrix stiffness in the fibrotic range in primary lung fibroblasts (17, 34, 63). However, S100A4’s effects have been shown to be cell type- and context-specific, partly explained by its varied downstream interacting proteins, and by its varied location, whether nuclear, cytoplasmic, or extracellular (29, 36, 46, 64, 65, 66, 67, 68, 69). We show that S100A4 in primary MLFs remains in the cytoplasm under basal conditions and in response to either increased stiffness or TGFꞵ. These findings parallel the cytoplasmic S100A4-NMII-A interaction seen in stage-specific embryonic antigen-4 (SSEA4+) stem cells from lungs of patients with IPF after 21 days using standard tissue culture conditions, although spontaneous cell motility, rather than myofibroblast differentiation, was enhanced under those conditions (67). Furthermore, secreted S100A4 by myeloid cells has been shown to result in pro-fibrotic phenotypic responses *in vitro*, albeit under standard tissue culture conditions using supra-physiologic substrate stiffness (45, 46). In this light, a critical advance in our work is that experiments mimicking secreted S100A4 failed to rescue the myofibroblast phenotype in S100A4 KO fibroblasts on pathophysiologic range substrate stiffness, demonstrating an absolute requirement for endogenous expression of S100A4 in fibroblasts. Thus, we show for the first time that intracellular fibroblast S100A4 is a critical regulator of the cytoskeletal response to pathophysiologic range substrate stiffness.

The NMII family of proteins is comprised of two common regulatory light chains and three individual isoform-defining heavy chains (A, B, and C). Prior work shows that S1004 selectively interacts with the NMII-A isoform and that NMII-A and II-B can co-assemble and have redundant roles within cells (41). Despite its abundance in the center of the cell and its upregulation with knockout of S100A4, we found that there is no incorporation of NMII-B into central, contractile actomyosin stress fibers, and therefore little resultant contractile force generation (Figs. 3*E* and 8). Prior work in purified protein systems suggests that regulation of NMII-A and NMII-B occurs through S100A4 binding or S1943 phosphorylation of the heavy chain, respectively (Vicente and Ecsedi refs). Our data show no changes in NMII-B distribution despite its increased expression in S100A4 KO cells. This finding is consistent with and extends prior work suggesting mutually exclusive regulation of NMII-A and NMII-B assembly/distribution in live cells (23, 70). Our data is thus consistent with the selective role of S100A4 on the NMII-A isoform and thereby further supports NMII-A’s critical role in the transition to a contractile myofibroblast (71).

### Lack of NMIIA-S100A4 interactions drives cytoskeletal changes *via* locking NMII in peripheral filaments

Work in our lab on normal lung fibroblasts (data not shown) and in others’ demonstrate a role for S100A4 in facilitating migration in multiple cell types, by modulating NMII-A turnover at the leading edge of the cell to promote forward protrusions and thereby enhance cell motility (31, 72, 73). In suspended cells, and during the initial spreading of adherent cells, actins organize in a cortical shell around the cell periphery, and with adherence, they actively reorganize their cytoskeletal structures to match the force exerted by substrate stiffness (74, 75, 76). Whereas cortical stress fibers assemble *de novo* from the actin pool independent of myosin, it has been shown that NMII drives the assembly of actin filaments and/or central stress fiber-focal adhesion complexes to generate force (77, 78). Therefore, not only do stress fibers and focal adhesions play a major role in the cell’s response to matrix biophysical cues, but NMII is critical for the initiation of the response. Our current work demonstrates, that in the absence of endogenous fibroblast S100A4, NMII-A fails to accumulate in the cell center, and thereby cannot act as a nidus for the development of contractile actomyosin filaments. This is robustly demonstrated by the absence of both actin and NMII-A filament formation under conditions of pathophysiological-range substrate stiffness in the S100A4 KO fibroblasts, in concert with the persistence of NMII-A filaments in a cortical, ring-like structure (Fig. 3*C*, bottom row, 8–25 kPa). The prolonged t_1/2_ of filamentous NMII-A noted on FRAP analysis, and the slowed decay of actin fibers selectively in the cell periphery of S100A4 KO fibroblasts demonstrate the importance of S100A4 for actomyosin filament turnover. This impaired actomyosin cortical fiber turnover upon loss of S100A4 was noted in response to only pathophysiological-range substrate stiffness *in vitro*, but also selectively on actual fibrotic lung tissue explants. These novel findings can be explained by the previously characterized effect of calcium-activated S100A4 to bind to and unravel the heavy chain of filament-tethered NMII-A, leading to filament disassembly and refolding of NMII-A monomers into the freely diffusible 10S conformation (34). Thereafter, upon kinase/phosphatase-dependent activation of the regulatory light chain (*i.e.*, by ROCK, MLCK or inhibition by MLCP), the 10S NMII-A monomers unfurl and spontaneously re-assemble along with actin to form force-generating actomyosin filaments in a different location (23). This newly localized, filamentous actomyosin can then mediate the local cytoskeletal remodeling that supports the cellular protrusions necessary for cell migration, and/or initiate the development of central, contractile stress fibers necessary for myofibroblast transdifferentiation (31, 72). As the total cellular pool of filamentous actin and NMII-A is limiting, our findings of preferential locking of actomyosin filaments in the cell cortex would lead to a relative paucity of NMII-A in the cell center, as we detected. The lack of central NMII-A underlies the failure of assembly and function of the central stress fiber contractile apparatus we observed. Similarly, biochemical evidence of over-exuberant assembly of peripheral NMII-A and actin filaments was also noted in macrophages lacking S100A4, resulting in impaired chemotaxis to CSF-1 (79).

Historically, enhanced expression of α-SMA has been a key defining hallmark of myofibroblast transdifferentiation (44). However, recent data from murine models and single cell analyses of IPF patient lungs indicates that there is significant heterogeneity among fibroblast populations, with substantive, but partial, overlap among collagen-, α-SMA- and S100A4-expressing populations (80, 81, 82, 83, 84). Our data and others’ document that fibroblast co-expression of S100A4 and α-SMA, along with α-SMA’s incorporation into stress fibers in pro-fibrotic fibroblasts, is both context (*e.g.*, substrate stiffness) and cell-type-dependent (29, 36, 69). We further show that in the absence of S100A4, focal adhesions are unable to mature, as shown by their reduced size and absence of vinculin. These immature adhesions are incapable of supporting the development of the myofibroblast, and they fail to support tension-dependent recruitment of α-SMA into stress fibers. As a result, our data reveal that fibroblasts lacking S100A4 are unable to generate the traction force necessary to stabilize the cell when interacting with pathophysiologic-range substrate stiffnesses. Taken together, these data demonstrate that endogenous fibroblast S100A4 underlies several critical cytoskeletal remodeling steps whereby stiffness in the pathophysiological range mediates fibroblast-to-myofibroblast transdifferentiation.

### Effects of S100A4 on homeostasis and disease pathophysiology

Using additional robust, quantitative, physiologic, and clinically relevant readouts, our work confirms and extends prior work showing that global KO or pharmacologic inhibition of S100A4 protects against bleomycin-induced pulmonary fibrosis in mice (45, 46). We further show for the first time that both mRNA and protein levels of S100A4 are upregulated in human IPF fibroblasts, and that S100A4 plays a role in myofibroblast transdifferentiation in lung fibroblasts interacting with actual human fibrotic lung tissue explants. In addition to pulmonary fibrosis, S100A4 has been implicated in fibrosis in the liver, heart, skin, and kidney (27, 28, 29, 30). Furthermore, endogenous cancer-associated fibroblast S100A4 functions to induce resistance to chemotherapeutic agents through myofibroblast transdifferentiation and resultant stiffening of the tumor stromal matrix (85). Additionally, interaction of NMII-A with S100A4 has been mechanistically implicated in cancer metastasis (31, 32, 33). Thus, our mechanistic work supports the further development of previously-identified small molecule inhibitors of S100A4 for the amelioration of disease.

### Limitations

Although we show a robust effect of S100A4 on stiffness-dependent myofibroblast transdifferentiation through redistribution of NMII-A using several complementary measures, this study has some limitations. While knocking out S100A4 in normal lung fibroblasts largely abrogates myofibroblast transdifferentiation, we cannot rule out the possibility that other S100 isoforms contribute to transdifferentiation in IPF fibroblasts (70). The property of stiffness is only one of several matrix cues that fibroblasts receive *in vivo.* Varying matrix protein type and density, as well as a 3-dimensional environment, have all been shown to affect fibroblast phenotype (86), and these properties were not examined in this study. However, the finding of impaired myofibroblast transdifferentiation on actual fibrotic lung matrix somewhat mitigates this concern. The protective effects of global KO of S100A4 on *in vivo* fibrosis in live mice that we observe do not discern between the effects of endogenous or secreted S100A4, or specifically identify the relevant cell subtype. Hopefully, emerging new single-cell data will support specific targeting of fibroblast and macrophage cell subtypes.

### Summary

In summary, as shown in the proposed schematic model (Fig. 11), our study reveals for the first time a key mechanoeffector role of endogenous fibroblast S100A4 in lung fibrogenesis *in vitro* and *in vivo*. Through loss- and gain-of-function studies we show that S100A4 is upregulated and facilitates redistribution of NMII-A from the periphery to the center of the cell, in a stiffness- and calcium-dependent manner, allowing the development of contractile, central actomyosin stress fibers and myofibroblast transdifferentiation. Our data suggest that the cryptic substrate stiffness-dependent effects of S100A4 on cytoskeletal remodeling should be considered when interpreting *in vitro* studies performed on supra-physiologically stiff substrates such as tissue culture plastic. Collectively, this mechanistic work provides strong support for specifically targeting the ECM stiffness-S100A4-NMII-A axis to halt fibrosis progression in IPF, and potentially a number of other fibrotic and malignant disorders.

## Experimental procedures

### Materials

Bleomycin sulfate and antibodies to α-SMA and vinculin were obtained from Sigma Aldrich. Anti-FSP1/S1004 antibody was obtained from Millipore. Alexa Fluor-phalloidin, ProLong Gold Antifade Reagent, and Alexa-Fluor conjugated secondary antibodies were obtained from Invitrogen. Antibodies to myosin IIA, myosin IIB, and phosphomyosin light chain 2 (p-MLC, Thr-18/Ser-19) were obtained from Cell Signaling Technology (Beverly, MA). Rabbit IgG was obtained from Jackson Immunoresearch. Normal mouse IgG and TGFβ1 was obtained from R&D Systems. Antibody to GAPDH was obtained from Fitzgerald, Lamin A-C was obtained from BD Biosciences, and type 1 collagen was obtained from Southern Biotech. Recombinant mouse S100A4 (ab109341) was obtained from Abcam. SiR-actin (CY-SC001) was obtained from Cytoskeleton, Inc. Glass-bottom or plastic plates (12- or 24-well), 35 mm and 100 mm dishes, and 12 mm coverslips containing activated polyacrylamide gels of 1 kPa, 8 kPa, 25 kPa were custom-made by Matrigen Life Technologies. Soft Trac 25 kPa polyacrylamide gels containing 1 μm fluorescent yellow-green spheres used for traction force microscopy were obtained from Matrigen Life Technologies. C57Bl/6 mice were obtained from The Jackson Laboratory. S100A4 KO mice were kindly provided by Dr Anne Bresnick. Primary MLFs were derived from 7- to 10-week-old wild type (WT) or S100A4 KO mice and propagated in complete media (MEM supplemented with 10% fetal bovine serum) as described previously (17). Normal human lung fibroblasts (NL HLF, 19Lu, passages 4–7) were obtained from ATCC and maintained and propagated as previously described (17).

### Western Blot analysis

Immunoblotting was performed as previously published with minor modifications (87). For S100A4 detection, cell lysates were separated on 16.5% tris-tricine SDS-PAGE Criterion gels (BioRad) and transferred to 0.22 micron PVDF membranes (ThermoFisher). For NMII-A or NMII-B detection, cell lysates were separated on either 7.5% (Criterion) or 4 to 15% gradient mini-gels (BioRad) SDS-PAGE gels and transferred to 0.45 micron PVDF Membranes (Thermo Scientific). Primary and HRP-tagged secondary antibodies were used as published and detected using ECL Prime (Cytiva) as published (14, 17, 48). Band density was directly measured on a UVP Spectrophotometer and normalized to either that of actin or GAPDH.

### Nuclear and cytoplasmic extraction

Nuclear and cytoplasmic extracts were separated and prepared using the NE-PER kit (Thermo Scientific) according to the manufacturer’s instructions. Briefly, MLFs were plated on to varying stiffness fibronectin-coated substrates and serum-starved overnight, followed by the addition of TGFβ (10 ng/ml) for 48 h. Cells were trypsinized and separated into pellets and supernatants by centrifugation and using the kit reagents. Protein concentrations were determined by BCA assay and samples were loaded in equal protein amounts onto 7.5% Criterion TGX (BioRad) gels. Immunoblotting was performed as described above. GAPDH was used as a cytoplasmic loading control, and Lamin A/C was used as a nuclear loading control.

### Cell responses to varying stiffness ECM

Custom made 12-well glass-bottom plates (for immunofluorescence) and plastic-bottom plates (for immunoblotting) containing activated polyacrylamide gels of 1 kPa, 8 kPa, 25 kPa, and glass (Matrigen Life Technologies) were coated with 1 μg/ml fibronectin for 2 h (37 °C) and cells were allowed to attach for indicated times, as described previously (17). TGFβ and/or recombinant mouse S100A4 at concentrations and time points indicated for each experiment was added, followed by the described analyses.

### Immunolabeling and confocal microscopy

To determine peripheral *versus* central regions of the cell, fibroblasts were seeded onto the appropriate substrate, and maintained in MEM supplemented with 1% FBS for 24 h. Cells were then transferred to 1% BSA in serum-free media ± TGFβ (at indicated concentrations and times). The attached cells were fixed with 4% paraformaldehyde, permeabilized by 0.05% Triton X-100, and blocked with 5% normal goat serum. To label the α-SMA-incorporated cytoskeletal fibers, fibroblasts were incubated with primary anti-α-SMA antibody (1:1000) followed by Alexa Fluor-488 secondary antibody (1:500), while in some cases, F-actin was stained with Alexa Fluor 594-phalloidin (1:40). Images were captured using an inverted Leica SP8 confocal microscope with a 20x/0.4NA long working distance objective. Using ImageJ (NIH), a line was drawn across the cell in an area excluding the nucleus and a plot profile of the intensity along the line was created. On the plot profile, the area of each peripheral peak and three peaks representative of central cell intensities was calculated. The peripheral and central intensities were averaged and used to calculate the ratio. One plot profile was created for each cell and multiple cells were analyzed per field.

### Fluorescence recovery after photobleaching (FRAP)

WT and S100A4 KO MLFs were plated onto fibronectin-coated glass coverslip-bottom dishes and transfected with the EGFP NMII-A fusion protein expressing plasmid, pEGFP-C3-NMHC-IIA (kindly provided by Dr Anne Bresnick (88)). After 6 h, transfection media was replaced with 10% SCM for 48 h. FRAP experiments were performed on an inverted Leica SP8 confocal microscope. Ten sequential frames of the cell of interest were taken prior to bleaching. The bleaching was performed using a photo activation laser of wavelength 488 nm at 63x magnification. Two circular regions (diameter 2 μm) were chosen in the periphery and central region of each cell and bleached ten times (0.865 s each, total 7.783 s) without delay by using 488 nm laser at 100% intensity. Recovery after bleaching was recorded for 5 min in 10 s intervals. Data for each cell was exported to Microsoft Excel. EasyFRAP software (http://easyfrap.vmnet.upatras.gr/) was used to generate a raw data fluorescence recovery curve after correction for background fluorescence and total cell fluorescence loss. The quality of the data was assessed using bleach depth and gap ratio. Curves were normalized using a double normalization equation. The normalized data was fit to the FRAP equation I(t) = A-Ae^−τt^, for which I(t) equals the fluorescence intensity as a function of time, A equals the plateau fluorescence intensity, τ is equal to the dissociation rate, the halftime of recovery is equal to −*ln*0.5/Iτ, and the mobile fraction is equal to 1-A. Only curves with a residual fit R-squared greater than 0.5 were used for analysis.

### Actin dynamics assay

WT or S100A4 KO fibroblasts were plated on fibronectin-coated 25 kPa gels in a 35-mm glass bottom dish (Matrigen) and serum-starved in 1% BSA/SFM for 4 h. SiR-Actin (SA; 75 nm) was added for either the entire experiment or for 4 h then washed off. TGFꞵ (2 ng/ml) was added and the live cells were imaged using a Leica SP8 inverted confocal microscope with a 20x/0.4NA long-working distance objective (24 h, images taken every 30 min for persistent SA; 6 h, images taken every 15 min when SA not present, 37 °C, 5% CO_2_, 95% humidity).

### Traction force microscopy

Polyacrylamide substrates (shear moduli 25 kPa) embedded with 0.2 μm yellow/green fluorospheres (Matrigen) were incubated with 10 ug/ml fibronectin for 2 h. Wild-type MLFs or S100A4 KO MLF were plated on the gels and allowed to attach in 10% serum-containing media for 1 h. The media was aspirated and replaced with 1% BSA in serum free media ± 2 ng/ml TGFβ for 24 h. Images of gel surface-conjugated fluorescent beads were acquired from ten different locations before and after trypsinization using a Leica DMI6000 inverted microscope (Leica Microsystems) equipped with a Leica 7000T camera and LAS-X software at 10x/0.4NA. Bead displacement and resultant traction force were calculated using TractionsForAll v1.0 software (Mayo Clinic) as previously described (89, 90, 91, 92).

### Lentiviral constructs

Lentiviral constructs expressing control EGFP, WT S100A4-GFP LV, and mutant S100A4 (mut-S100A4-GFP) were designed by Dr Anne Bresnick (93) and produced by VectorBuilder. For transfection, subconfluent WT or S100A4 KO MLF monolayers were exposed to 100 MOI of one of the above lentiviral constructs overnight in a complete MEM medium supplemented with polybrene (2 μg/ml, Santa Cruz Biotechnology). The following morning, LV-containing media was aspirated, the cells were washed twice with warm serum-free media (SFM), and then maintained in complete media for 48 h. The cells were then serum-starved in SFM + 1% BSA for 4 h, then the media was replaced with SFM + 1% BSA ± TGFβ (2 ng/ml) for 24 h. Cells were then fixed and fluorescently stained as described above and previously published (14, 17, 48).

### Bleomycin-induced pulmonary fibrosis model

Induction of pulmonary fibrosis in S100A4 KO 8 to 12 week old mice (generated by Dulyaninova *et al.* (79)) and age-matched female congenic WT C57BL/6 mice was performed by oropharyngeal instillation of bleomycin (2 U/kg) or phosphate-buffered saline (as a control), as previously published (94). Twenty-one days after bleomycin treatment, the lungs were inflated with OCT (Sakura Finetek) and ten micron sections were stained with trichrome. Compliance measurements were done using an animal ventilator (Scireq) equipped with software (FlexiVent, Scireq) to record and analyze the measurements. Anesthetized, tracheostomized, paralyzed, and mechanically ventilated mice were used during all static/dynamic P-V loop measurements (as a measure of lung compliance). Lung compliance was taken as the slope of the deflation curve just above FRC, as published (14). Collagen deposition was quantified biochemically by measuring hydroxyproline levels or detecting collagen-1 in the lung tissue extracts by immunoblotting analysis (95). α-SMA, NMII-A, and NMII-B expression were analyzed by immunoblots of whole lung lysate extracts. For assessment of lung tissue interaction with fibroblasts, 10-μm sections of OCT-inflated lung from bleomycin (2 U/kg, 2 weeks) or saline-instilled control lung tissue were used as published previously (14, 17). MLFs were allowed to attach on the 5% BSA-blocked lung sections, and unattached cells were washed off with PBS. Lung section-containing cells were then labeled for the indicated proteins and imaged with confocal microscopy as published previously (17). Image J was used to create intensity plot profiles for each cell to quantify central: peripheral intensities, as described above.

### PCR

RNA was isolated from lung explant fibroblasts from IPF and normal patients using the Qiagen RNAEasy kit per the manufacturer’s instructions. Quantitative real-time PCR (qPCR) was conducted using the ABI Prism 7300 real-time PCR System (Applied Biosystems, Life Technologies). The following specific primers for S100A4 were custom-designed using Integrated DNA Technologies (IDT, Coralville, IA):

human S100A4 fwd – GATGAGCAACTTGGACAGCA

human S100A4 rev – ACTCTTGGAAGTCCACCTCGT

human GAPDH fwd – ACCACAGTCCATGCCATCAC

human GAPDH rev – TCCACCACCCTGTTGCTGTA.

The housekeeping gene GAPDH was used to normalize for the input of loaded cDNA. 1 μl of cDNA was mixed with the appropriate 100 nmol/ml primers and 2× SYBR Green Master Mix (Applied Biosystems, Life Technologies) in a total volume of 20 μl. Each qPCR reaction was carried out in a 96-well plate in duplicate with the following program: 95 °C for 10 min for initial denaturation, 40 cycles of amplification as follows (1): denaturation at 95 °C for 15 s (2), annealing and elongation at 60 °C for 1 min. Melting curve analysis was also done with a continuous temperature increasing from 60 °C to 95 °C with a rate of 0.1 °C/s to assess the specificity of the amplification process. Relative gene expression levels were calculated using the comparative Ct (ΔΔCt) method.

### Statistics

All data are expressed as mean ± SEM unless otherwise indicated. Statistical comparisons between control and experimental groups were performed with the Student’s t-tests or 1-way ANOVA test using SigmaPlot software. ANOVA test followed by the Student-Newman-Keuls test, Tukey Test, or Dunnet’s (*versus* control) was used for multiple comparisons. For comparisons between categorical variables, the Chi-square test was used. *p* <0.05 was considered significant.

### Study approval

All animal protocols were performed according to guidelines approved by the Cleveland Clinic Institutional Animal Care and Use Committee.

## Data availability

All data are contained within the manuscript and will be shared upon request.

## Supporting information

This article contains supporting information.

## Conflict of interest

The authors declare that they have no conflicts of interest with the contents of this article.

## Supporting information

Supplemental Video 1a FRAP of NMMII-A in peripheral *versus* central regions of WT MLFs. Confocal time lapse videos (63x/1.32NA orig. mag.) of recovery after photobleaching (FRAP) in the peripheral (red arrows) and central (yellow arrows) regions of WT fibroblasts. Images containing the entire cell, including the 2 μm diameter bleached regions of interest, were taken every 10 s for 5 min. **Supplemental Video 1b**. FRAP of NMMII-A in peripheral *versus* central regions of S100A4 KO MLFs. Confocal time lapse videos (63x/1.32NA orig. mag.) of recovery after photobleaching (FRAP) in the peripheral (red arrows) and central (yellow arrows) regions of S100A4 KO fibroblasts. Images containing the entire cell, including the 2 μm diameter bleached regions of interest, were taken every 10 s for 5 min. **Supplemental Video 2a**. Actin dynamics in WT MLFs in response to 25 kPa and TGFβ. Confocal time lapse videos (40x/1.25NA orig. mag.) of WT fibroblasts labeled with the fluorogenic live-imaging actin probe SiR-actin (SA). Images were taken every 30 min for 24 h following TGFꞵ (2 ng/ml) administration, under continuous SA labeled conditions. **Supplemental Video 2b**. Actin dynamics in S100A4 KO MLFs in response to 25 kPa and TGFβ. Confocal time lapse videos (40x/1.25NA orig. mag.) of S100A4 KO fibroblasts labeled with the fluorogenic live-imaging actin probe SiR-actin (SA). Images were taken every 30 min for 24 h following TGFꞵ (2 ng/ml) administration, under continuous SA labeled conditions. **Supplemental Video 2c**. Actin dynamics in WT MLFs in response to 25 kPa and TGFβ. Confocal time lapse videos (40x/1.25NA orig. mag.) of WT fibroblasts labeled with the fluorogenic live-imaging actin probe SiR-actin (SA). Images were taken every 30 min for 24 h following TGFꞵ (2 ng/ml) administration, under conditions of pulse (4 h) followed by washout of SA. **Supplemental Video 2d**. Actin dynamics in S100A4 KO MLFs in response to 25 kPa and TGFβ. Confocal time lapse videos (40x/1.25NA orig. mag.) of S100A4 KO fibroblasts labeled with the fluorogenic live-imaging actin probe SiR-actin (SA). Images were taken every 30 min for 24 h following TGFꞵ (2 ng/ml) administration, under conditions of pulse (4 h) followed by washout of SA.
